# Matrix-bound nanovesicles alleviate particulate-induced periprosthetic osteolysis

**DOI:** 10.1126/sciadv.adn1852

**Published:** 2024-10-18

**Authors:** Runzhi Liao, Marley J. Dewey, Jiayang Rong, Scott A. Johnson, William A. D’Angelo, George S. Hussey, Stephen F. Badylak

**Affiliations:** ^1^ McGowan Institute for Regenerative Medicine, University of Pittsburgh, Pittsburgh, PA 15219, USA.; ^2^ Xiangya Hospital, Central South University, Changsha, Hunan 410008, China.; ^3^ Department of Surgery, School of Medicine, University of Pittsburgh, Pittsburgh, PA 15219, USA.; ^4^ Department of Pathology, School of Medicine, University of Pittsburgh, Pittsburgh, PA 15219, USA.; ^5^ Department of Bioengineering, University of Pittsburgh, Pittsburgh, PA 15219, USA.

## Abstract

Aseptic loosening of orthopedic implants is an inflammatory disease characterized by immune cell activation, chronic inflammation, and destruction of periprosthetic bone, and is one of the leading reasons for prosthetic failure, affecting 12% of total joint arthroplasty patients. Matrix-bound nanovesicles (MBVs) are a subclass of extracellular vesicle recently shown to mitigate inflammation in preclinical models of rheumatoid arthritis and influenza-mediated “cytokine storm.” The molecular mechanism of these anti-inflammatory properties is only partially understood. The objective of the present study was to investigate the effects of MBV on RANKL-induced osteoclast formation in vitro and particulate-induced osteolysis in vivo. Results showed that MBV attenuated osteoclast differentiation and activity by suppressing the NF-κB signaling pathway and downstream NFATc1, DC-STAMP, c-Src, and cathepsin K expression. In vivo, local administration of MBV attenuated ultrahigh molecular weight polyethylene particle-induced osteolysis, bone reconstruction, and periosteal inflammation. The results suggest that MBV may be a therapeutic option for preventing periprosthetic loosening.

## Teaser

Matrix-bound nanovesicles mitigate osteoclast formation and particle-induced periprosthetic osteolysis in vitro and in vivo.

## INTRODUCTION

Total joint arthroplasty is one of the most common interventions for end-stage arthritic or traumatic joints, with an estimated 63,500 primary total hip arthroplasty (THA) and 1.26 million primary total knee arthroplasty (TKA) procedures to be performed annually in the U.S. by 2030, and a 10-year revision rate of approximately 12% (*1*, *2*). Despite advancements in the biophysical properties of implants, prolonged and repeated mechanical loading and thermodynamically driven electrochemical processes can cause the release of metallic and ultrahigh molecular weight polyethylene (UHMWPE) wear debris ranging in size from nano- to micrometers (*3*). Phagocytosis of these particles by tissue-resident macrophages results in chronic inflammation and osteoclastogenesis via receptor activator of nuclear factor κΒ (NF-κB) ligand (RANKL)/RANK axis signaling. Nondigestible particles repeatedly stimulate monocyte/macrophages to differentiate into osteoclasts leading to periprosthetic osteolysis (PPOL) in the short term, aseptic loosening (APL) in the long term, and eventual prosthetic failure requiring revision (*4*, *5*). Surgical correction of APL accounts for 55% of THA revisions and 30% of TKA revisions, and as the number of joint replacement procedures increase, there is a growing need for an effective treatment for PPOL (*6*).

Extracellular matrix (ECM)–based biomaterials provide a biologically compatible scaffold for tissue repair and regeneration and have been used in diverse medical applications (*7*–*11*). The ECM is a three-dimensional meshwork of structural and functional macromolecules that are remodeled by and provide biophysiochemical feedback to resident cells to maintain homeostasis (*7*, *12*, *13*). Acellular ECM and its derivatives have been shown to have immunomodulatory properties, largely effected through modulation of macrophage phenotype, and have produced promising results in preclinical models of inflammatory diseases (*8*, *9*, *14*–*23*). Moreover, intra-articular injection of urinary bladder matrix (UBM) reduces osteoarthritis and NF-κB1 expression (*24*). Recent studies have shown that matrix-bound nanovesicles (MBVs) are a key mediator of the immunomodulatory effects of ECM (*25*, *26*). MBVs are a recently described class of extracellular vesicle (EV) integrally bound to the ECM, with unique composition, cargo, and function distinct from those of other EV types (*25*–*30*). MBVs were shown to modulate macrophage phenotype and prevent adverse bone remodeling in a preclinical model of rheumatoid arthritis (RA) (*29*). However, the effect of MBV on osteoclast differentiation and function has not been explored (*4*, *5*, *27*, *29*).

In the present study, RANKL-treated RAW264.7 monocyte/macrophages were used as an in vitro model of osteoclastogenesis, and a UHMWPE particulate-induced calvarial osteolysis murine model was used to show that MBVs inhibit osteoclast differentiation by inhibiting downstream NF-κB signaling with associated attenuation of UHMWPE-induced soft-tissue inflammation, osteolytic destruction, and remodeling in vivo.

## RESULTS

### MBV characterization and assessment of cytocompatibility

**Fig. 1.**
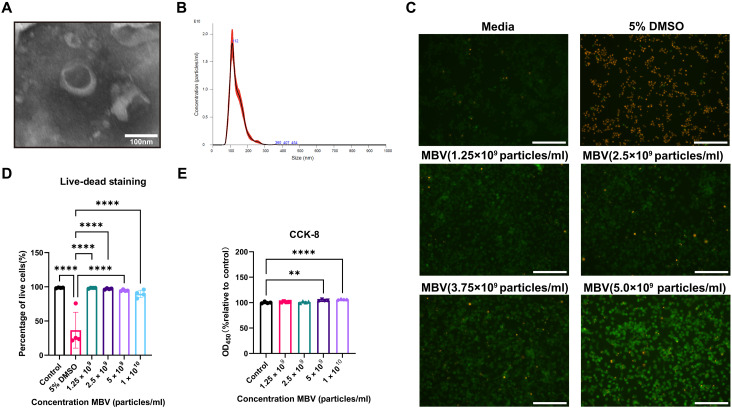
MBV characterization and assessment of cytocompatibility. (**A**) TEM imaging of MBV isolated from porcine urinary bladder matrix (UBM). Scale bar, 100 nm. (**B**) MBV size as measured by nanoparticle tracking analysis (NTA). (**C**) RAW264.7 cells were treated with phosphate-buffered saline (PBS), 5% dimethyl sulfoxide (DMSO), and serial dilutions of MBV ranging from 1.25 × 10^9^ to 1 × 10^10^ particles/ml (MBV versus cells ratio 1.25 × 10^5^ to 1 × 10^6^ particles/ml per cell) for 24 hours and assayed for cytotoxicity by live/dead staining. Representative fluorescence images. Scale bars, 100 μm. (**D**) Percentage of live cells. PBS control, 5% DMSO, and various doses of MBV. Values are shown as mean ± SD (*n* = 4). Significant differences were determined as *P < 0.05. (**E**) Cell proliferation and cytotoxicity were assessed by cell counting kit 8 (CCK-8) assay. Values are shown as mean ± SD (*n* = 4). Significant differences were determined as *P < 0.05. OD_450_, optical density at 450 nm.

Conventional identification of EVs includes morphology, particle size distribution, and surface marker molecule identification. However, no characteristic exosomal marker that distinguishes it from other EVs has yet been identified for MBV (*31*, *32*). MBVs were isolated from porcine UBM and characterized by transmission electron microscopy (TEM) (Fig. 1A) and nanoparticle tracking analysis (NTA) (Fig. 1B). The results show that MBV had an average diameter of 112 nm and classic concave spherical EV morphology under TEM. Following MBV treatment of RAW 264.7 macrophages, there was no significant difference in cytotoxicity compared to phosphate-buffered saline (PBS) control based on live-dead staining across serial dilutions of MBV ranging from 1.25 × 10^9^ to 1 × 10^10^ particles/ml (*P* >0.05; Fig. 1, C and D). Treatment with 5% dimethyl sulfoxide (DMSO) induced a significant decrease in cell viability compared to PBS control and compared to all dilutions of MBV based on live-dead staining (*P* < 0.05; Fig. 1, C and D). For cell proliferation and cytotoxicity, different concentrations of MBV were cocultured with macrophages for 24 hours. There was no significant difference in cell proliferation between the 1.25 × 10^9^ particles/ml group, 2.5 ×10^9^ particles/ml group, and the PBS control group (*P* >0.05; Fig. 1E). However, the 5 × 10^9^ particles/ml group and 1 × 10^10^ particles/ml groups each caused a significant increase in cell proliferation by approximately 5% compared to PBS control group (*P* < 0.05; Fig. 1E). Results show that MBVs are not cytotoxic to RAW 264.7 cells.

### Macrophages uptake MBV and MBVs reduce RANKL-induced osteoclast formation and activity in vitro

**Fig. 2.**
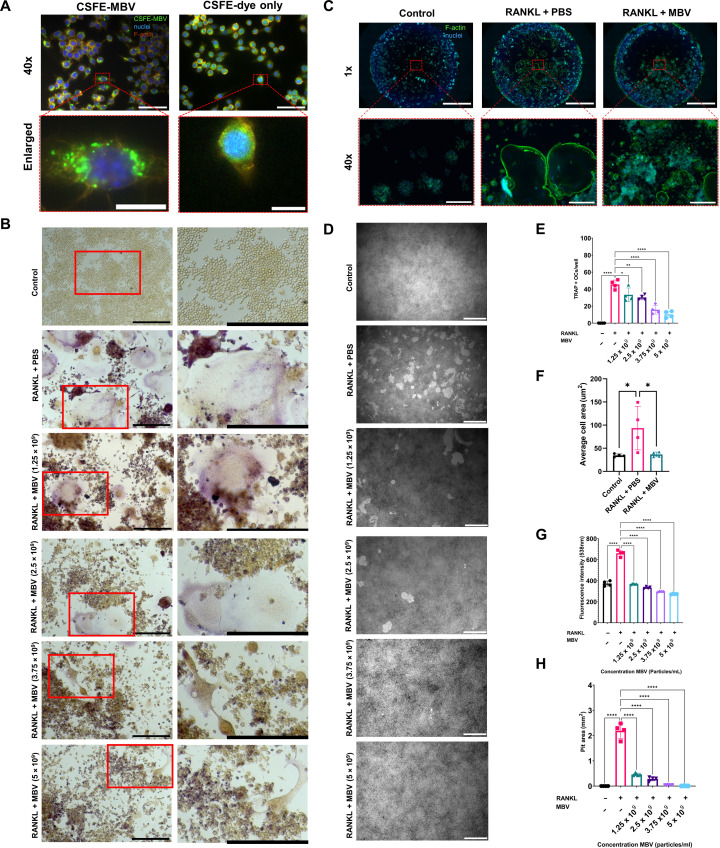
MBVs mitigate RANKL-induced osteoclast formation and activity in vitro. (**A**) Cellular uptake of MBV by RAW 264.7 cells. CFSE-labeled (green) MBV were added to the RAW 264.7 cells culture media for 1 hour; nuclei were labeled with 4′,6-diamidino-2-phenylindole (DAPI; blue), and cytoskeleton F-actin was labeled with 594-phalloidin (orange) before imaging. Representative IF images (40× and enlarged). Scale bars for 40× images, 50 μm; enlarged images, 10 μm. (**B**) MBVs reduce osteoclasts differentiation. RAW 264.7 cells were cultured with RANKL (30 ng/ml) and serial doses of MBV ranging from 0 to 5 × 10^9^ MBV/ml for 5 days. Osteoclasts were determined with tartrate-resistant acid phosphatase (TRAP) staining. Representative light microscope TRAP staining images (10× and enlarged). Scale bars, 200 μm. (**C**) Raw 264.7 cells were cultured with RANKL (30 ng/ml) with or without MBV for 5 days. Actin ring formation was determined with FITC-phalloidin, and nuclei were labeled with DAPI. Representative immunofluorescence images (1× and 40×). Scale bars for 1× images, 2000 μm; 40× images, 100 μm. (**D**) MBVs reduce osteoclasts activity. RAW 264.7 cells were cultured with RANKL (30 ng/ml) and serial doses of MBV ranging from 0 to 5 × 10^9^ MBV/ml on a calcium phosphate–coated plate for 7 days. Resorption areas were determined with a light microscope (5×). Scale bars, 500 μm. (**E**) Numbers of osteoclasts (TRAP positive, nuclei > 3). Values are shown as mean ± SD (*n* = 4). Significant differences were determined as *P < 0.05. (**F**) Average cell area (*n* = 4). Values are shown as mean ± SD (*n* = 4). Significant differences were determined as *P < 0.05. (**G**) At day 6, bone resorption assay culture media were tested for fluorescence intensity. Fluorescence intensity (*n* = 4). Values are shown as mean ± SD (*n* = 4). Significant differences were determined as *P < 0.05. (**H**) Resorption pit area (*n* = 4). Values are shown as mean ± SD (*n* = 4). Significant differences were determined as *P < 0.05.

MBVs were labeled with 5-(and-6)-carboxyfluorescein diacetate, succinimidyl ester (CFSE) and added to RAW 264.7 cells for 1 hour to examine MBV uptake. Nuclei were labeled with Hoechst and cytoskeleton F-actin labeled with 594-phalloidin before imaging (Fig. 2A). The effect of MBV on osteoclast formation was investigated by culturing RAW 264.7 cells with serial dilutions of MBV ranging from 0 particles/ml (PBS) to 5 × 10^9^ particles/ml in the presence of RANKL (30 ng/ml) for 5 days. The presence of multinucleated osteoclasts was identified by tartrate-resistant acid phosphatase (TRAP) staining (nuclei were identified by hematoxylin staining after TRAP staining; fig. S1). The RANKL + PBS group showed more TRAP-positive, multinucleated osteoclasts compared to the control group (Fig. 2B). Serial dilutions of MBV ranging from 1.25 × 10^9^ to 5 × 10^9^ particles/ml + RANKL group showed a dose-dependent decrease in TRAP-positive, multinucleated osteoclasts compared to the RANKL + PBS group (Fig. 2B). Following RANKL induction, osteoclast formation decreased significantly with increasing MBV concentration compared to the RANKL + PBS group (*P* < 0.05; Fig. 2E). The TRAP^+^ osteoclast number in the RANKL + 5 × 10^9^ particles/ml MBV group was not significantly different from the control group (*P* >0.05; Fig. 2E). The effects of MBV on osteoclast activity were assessed by fluorescein isothiocyanate (FITC)–phalloidin–labeled actin rings, which relates to the bone matrix sealing zone formed by osteoclast to secreted matrix metalloproteases and acids (*33*). RAW 264.7 cells were cultured with or without MBV (5 × 10^9^ particles/ml) and RANKL (30 ng/ml) for 5 days. Results showed a smaller actin ring (average cell area) formation in RANKL + MBV group compared to RANKL + PBS group (*P* < 0.05; Fig. 2, C and F). A fluorescence bone resorption assay assessed further osteoclast activity. RAW 264.7 cells were cultured with the same condition in the osteoclast formation test on a fluorescence-labeled calcium phosphate–coated plate for 7 days. At day 6, the culture media fluorescence intensity was assessed. At day 7, the cells were removed to assess the pit resorption area. Relative to RANKL + PBS group, MBV significantly decreased fluorescence intensity and pit resorption area (*P* < 0.05; Fig. 2, D, G, and H). Cumulatively, the results showed that MBVs reduce osteoclast formation and activity in vitro.

### MBVs mitigate RANKL-induced osteoclast formation by suppressing NF-kB signaling pathway in vitro

**Fig. 3.**
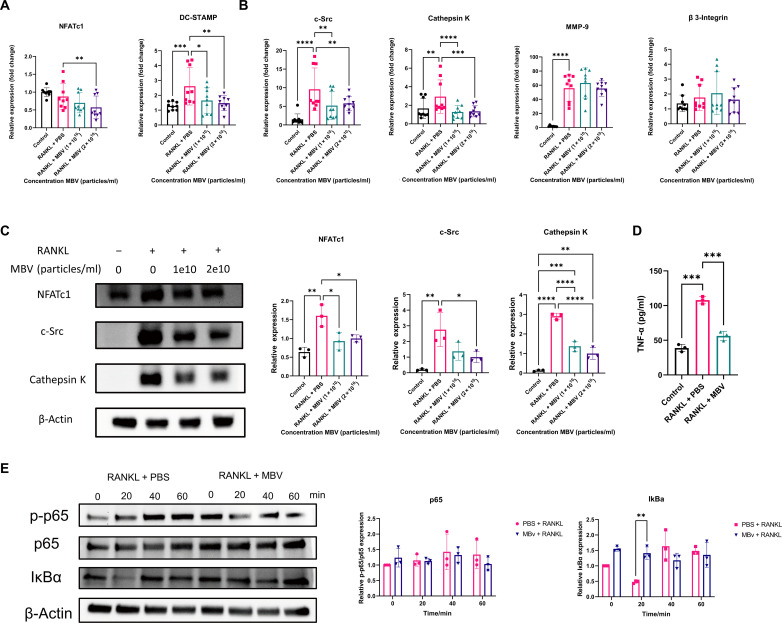
MBVs mitigate RANKL-induced osteoclast formation and functions by suppressing NF-κB pathway in vitro. RAW 264.7 cells were challenged with RANKL and various concentrations of MBV (1× 10^10^ to 2× 10^10^ particles/ml, MBV versus cells ratio 1× 10^4^ to 2 × 10^4^ particles/ml per cell), 1 day for osteoclast differentiation and activity–related gene RT-qPCR (A and B; RANKL, 50 ng/ml), 3 days for osteoclast differentiation and activity–related protein Western blot (C; RANKL, 50 ng/ml), and 5 days for TNF-α concentration in conditional media (D; RANKL, 30 ng/ml; MBV, 5× 10^9^ particles/ml). (**A**) Osteoclast differentiation regulators (NFATc1 and DC-STAMP) relative expression (2^−△△T^) were measured by RT-qPCR (values = means ± SD; *n* = 9; significant differences, *P < 0.05). (**B**) Osteoclast activity–related genes (cathepsinK, c-Src, MMP-9, and β3-integrin) relative expressions (2^−△△T^) were measured by RT-qPCR (values = means ± SD; *n* = 9; significant differences, *P < 0.05). (**C**) Osteoclast differentiation–related protein (NFATc1) and osteoclast activity proteins (c-Src and cathepsin K) relative expression was measured by Western blot (values = means ± SD; *n* = 3; significant differences, *P < 0.05). (**D**) TNF-α in conditional media from Raw 264.7 cells cocultured with RANKL with or without MBV for 5 days was determined by ELISA (values = means ± SD; *n* = 3; significant differences, *P < 0.05). (**E**) RAW 264.7 cells were challenged with RANKL (50 ng/ml) and MBV (2 × 10^10^ particles/ml) from 0 to 60 min. NF-κB pathway proteins were determined by Western blot. Representative Western blotting images of the effects of MBV on p-p65, p65, and IĸB-α. Quantification of the ratios of band intensity of p-p65/p65 and IĸBα relative to β-actin expression (values = means ± SD; *n* = 3; significant differences, *P < 0.05).

To determine the effect of MBV on initial osteoclast differentiation, expression of the master transcription factor *NFATc1* and the osteoclast precursor multinucleation factor dendritic cell-specific transmembrane protein (*DC-STAMP*) was measured by reverse transcription quantitative polymerase chain reaction (RT-qPCR) 24 hours after MBV treatment (*33*, *34*). The expression of *NFATc1* was significantly decreased after treatment with 2 × 10^10^ MBV/ml compared to the RANKL + PBS group (*P* < 0.05; Fig. 3A), although treatment with a lower concentration (1× 10^10^ particles/ml) did not cause a decrease. *DC-STAMP* expression was increased in the RANKL + PBS group compared to the control group (*P* < 0.05; Fig. 3A), and MBV treatment at either concentration decreased its expression. The effect of MBV on expression of genes related to osteoblast function, including cathepsin K, c-Src, matrix metalloproteinase–9 (*MMP-9*), and β3-integrin was examined (*35*–*38*). While RANKL treatment (RANKL + PBS group) increased cathepsin K, c-Src, and *MMP-9* gene expression compared to the control group (*P* < 0.05; Fig. 3B), the addition of MBV significantly down-regulated cathepsin K and c-Src expression compared to the RANKL + PBS group (*P* < 0.05; Fig. 3B). *MMP-9* expression was not different between RANKL + PBS and RANKL + MBV groups (*P* > 0.05; Fig. 3B). Across all groups, β3-integrin expression showed no significant difference (*P* > 0.05; Fig. 3B).

These trends were mirrored at the protein level, where RANKL-induced cells showed increased expression of NFATc1, c-Src, and cathepsin K at the 3-day timepoint (*P* < 0.05; Fig. 3C), and the addition of MBV significantly abrogated these increases. Similarly, treatment with RANKL for 5 days increased the tumor necrosis factor–α (TNF-α) concentration in the media compared to the control group, and this was significantly reduced with the addition of MBV (*P* < 0.05; Fig. 3D).

To determine whether MBV-mediated modulation of NF-κB pathway signaling is involved in the observed reduction of osteoclastogenesis, macrophages were pretreated with MBV for 2 hours and then treated with RANKL for 0 to 60 min before analysis of pathway components by Western blot. Although the ratio of phosphorylated p65 versus p65 showed no significant difference between the RANKL + PBS group and the RANKL + MBV group, a significant decrease in the level of inhibitor of NF-κBα (IκBα) was observed at the 20 min timepoint in the RANKL + PBS group, which was not observed in the RANKL + MBV group (*P* < 0.05; Fig. 3E).

### MBV biodistribution in a mouse model of particulate-induced calvarial osteolysis

**Fig. 4.**
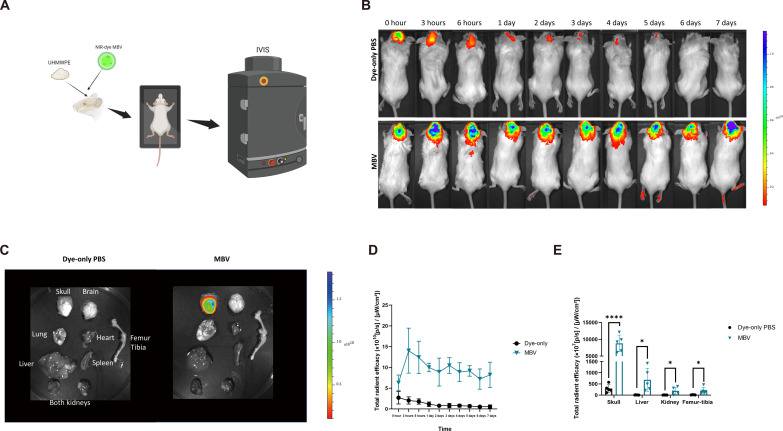
MBV biodistribution in mouse model of particulate-induced calvarial osteolysis. (**A**) Experiment design. Mice skull and periosteum were exposed to UHMWPE particles. Near-infrared (NIR)–labeled MBV pericalvarial administrated post-UHMWPE implantation surgery. From 0 hour to 7 days postsurgery, fluorescence signal was examined by an in vivo imagine system (IVIS). (**B**) Representative IVIS images from 0 hour to 7 days. (**C**) Seven days postinjection, mice were euthanized, and organs and bones were taken for IVIS imaging. Representative organs and bone IVIS images. (**D**) Total radiant efficacy ([p/s]/[uW/cm^2^]) of the calvarial region from 0 hour to 7 days (values = means ± SD; *n* = 5). (**E**) Total radiant efficacy ([p/s]/[uW/cm^2^]) of skull, liver, kidneys, and femur-tibia (values = means ± SD; *n* = 5; significant differences, *P < 0.05).

The biodistribution of near infrared-labeled MBV after local injection was examined in a mouse UHMWPE particulate-induced calvarial osteolysis model (Fig. 4A). In vivo imagine system (IVIS) imaging showed strong fluorescent signal accumulation in the pericalvarial region in the MBV group, while the dye-only PBS group showed a minimal fluorescent signal throughout the 7 days (Fig. 4B). The highest fluorescence signal in the MBV group was observed 3 hours postadministration, and the signal in this area did not decrease over 7 days (*P* > 0.05; Fig. 4, B and D). At 7 days postadministration, mice were euthanized and the calvarial bones, brains, heart, lungs, liver, kidneys, femurs, and tibias were removed to individually measure their fluorescent signal. Significantly higher signal was observed in the calvarial bones, liver, kidney, and femur/tibia in the MBV group relative to those in the dye-only PBS group (*P* < 0.05; Fig. 4, C and E), with the signal in calvarial bones in the MBV group approximately 20 times higher than that of other organs. Thus, pericalvarial administration of MBV resulted in a robust and lasting local accumulation and showed limited systematic distribution in a mouse UHMWPE particulate-induced calvarial osteolysis model.

### Local administration of MBV alleviates osteolysis and bone remodeling in UHMWPE particulate-induced osteolysis

**Fig. 5.**
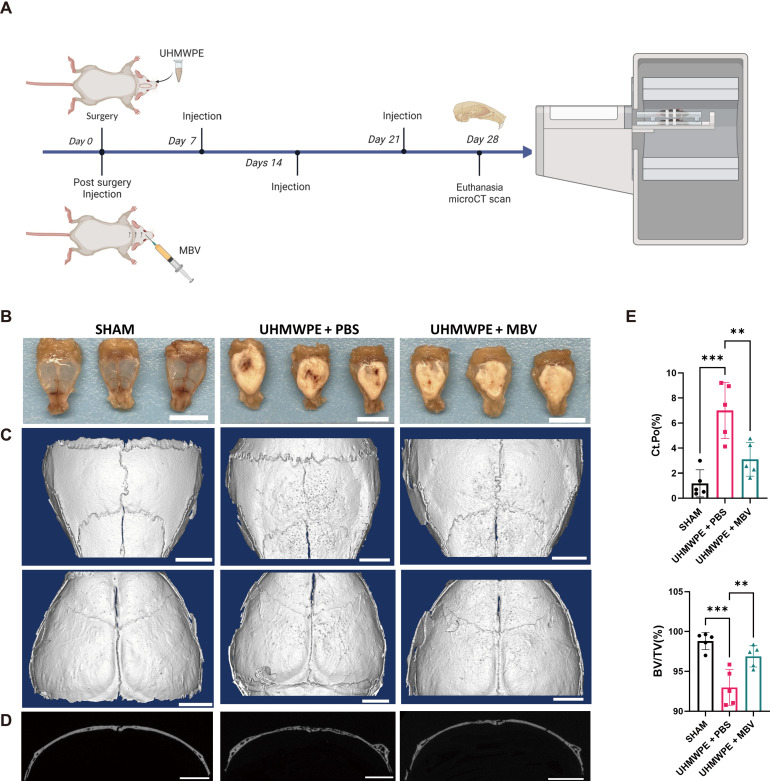
Local administration of MBV alleviates osteolysis and bone remodeling in mouse model of UHMWPE particulate-induced osteolysis. (**A**) Experimental design and treatment regimen. (**B**) Mice were euthanized to harvest skull specimens at day 28. Representative morphology images of mice skull. Scale bars, 10 mm. (**C**) Representative images of mice skull dorsal/ventral micro-CT imaging 3D reconstruction. Scale bars, 2 mm. (**D**) Representative images of coronal cross section of micro-CT scan of mouse skull. Scale bars, 2 mm. (**E**) Quantification of cortical porosity (Ct.Po) and bone volume versus tissue volume (BV/TV) of skull. (values = means ± SD; *n* = 5; significant differences, *P < 0.05).

On the basis of the IVIS results showing MBV retention in the pericalvarial area, osteolysis and bone remodeling by micro–computed tomography (micro-CT) following pericalvarial MBV treatment were examined (Fig. 5A). Gross observation of the calvarial bones following euthanasia showed dense, white tissue in the UHMWPE + PBS (vehicle control) group and the UHMWPE + MBV group relative to the sham group (Fig. 5B). Representative micro-CT three-dimensional reconstruction images showed multiple osteolytic areas in the dorsal and ventral sides of the calvarial bones in the vehicle control group, and the sagittal suture was obscured by extensive bone remodeling (Fig. 5C). In contrast, the MBV group showed fewer osteolytic areas and a preserved sagittal suture, comparable to the vehicle control group (Fig. 5C). Coronal cross-sectional micro-CT images showed nonphysiologic holes in the calvarial bone and loss of smooth, continuous bone surfaces in the vehicle control group, whereas the MBV group showed smooth, continuous bone surfaces with few osteolytic area (Fig. 5D). Last, quantification showed that cortical porosity (Ct.Po) was significantly increased and bone volume versus tissue volume (BV/TV) significantly decreased in the UHMWPE + PBS group relative to the sham group, but the addition of MBV resulted in a reversal of these trends (*P* < 0.05; Fig. 5E). These data suggest that pericalvarial administration of MBV alleviated osteolysis and bone remodeling in UHMWPE particulate-induced osteolysis.

### MBV alleviates inflammatory and osteoclastic activities in UHMWPE particulate-induced osteolysis

**Fig. 6.**
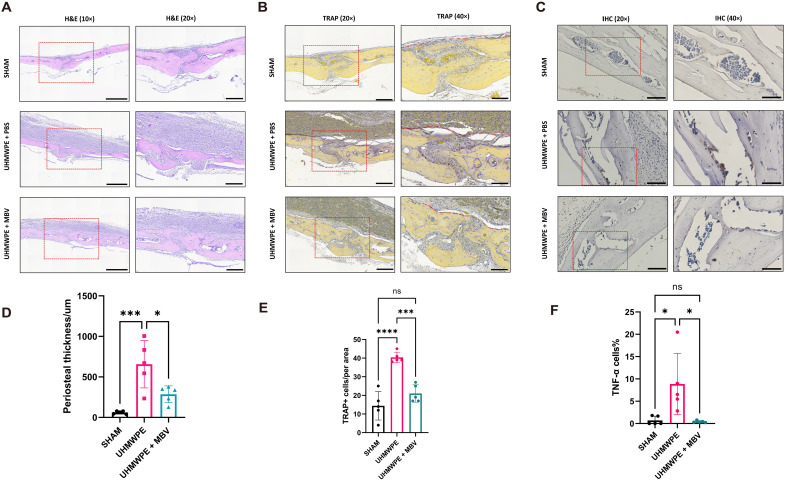
MBV alleviates inflammatory and osteoclastic activities in UHMWPE particulate-induced osteolysis. (**A**) Representative images of mice calvarial bone coronal paraffin sections (10× and 20×) hematoxylin and eosin (H&E) staining. Scale bars for 10× images, 500 μm; for 20× images, 200 μm. (**B**) Representative images of mice calvarial bone paraffin sections (20× and 40×) TRAP staining. Scale bars for 20× images, 200 μm; for 40× images, 100 μm. (**C**) Representative images of calvarial bone coronal paraffin sections (20× and 40×) TNF-α immunohistology [immunohistochemistry (IHC)] staining. Scale bars for 20× images, 100 μm; for 40× images, 50 μm. (**D**) Periosteal thickness of H&E staining images. Values are shown as mean ± SD (*n* = 5); significant differences, *P < 0.05. (**E**) TRAP^+^ cells numbers per 20× images. Values are shown as mean ± SD (*n* = 5); significant differences, *P < 0.05. (**F**) TNF-α cells percentage per 20× images. Values are shown as mean ± SD (*n* = 5); significant differences, *P < 0.05.

Histopathologic examination of the calvarial bone at 28 days post-UHMWPE implantation showed signs of severe inflammation and osteoclastic activity, including thickening of the periosteum, osteoclast formation, and cytokine expression compared to the sham group (sham versus UHMWPE + PBS; Fig. 6). Animals treated with MBV showed a reduction of periosteum thickness compared to the vehicle control group (*P* < 0.05; Fig. 6, A and D), with reduced TRAP^+^ osteoclast numbers (*P* < 0.05; Fig. 6, B and E) and fewer TNF-α^+^ cells (*P* < 0.05; Fig. 6, B and E). With MBV treatment, polarization of macrophages within periosteum toward M2 phenotype (*P* < 0.05; fig. S2). Thus, MBV alleviated inflammatory and osteoclastic activities in UHMWPE particulate-induced osteolysis in vivo.

## DISCUSSION

Therapies investigated for treatment of aseptic PPOL other than surgery have ranged from synthetic compounds to engineered exosomes (*39*–*42*). Strategies have focused on improvement of prosthesis materials and design and suppression of the inflammatory responses that lead to osteoclast differentiation and activity (*43*, *44*). Given the current inability to detect early aseptic PPOL, the limited treatment options for APL, and the increasing numbers of joint replacements, the need for therapies that target local foreign body–induced inflammation and osteoclast formation is unmet and clinically relevant (*1*, *2*, *6*).

**Fig. 7.**
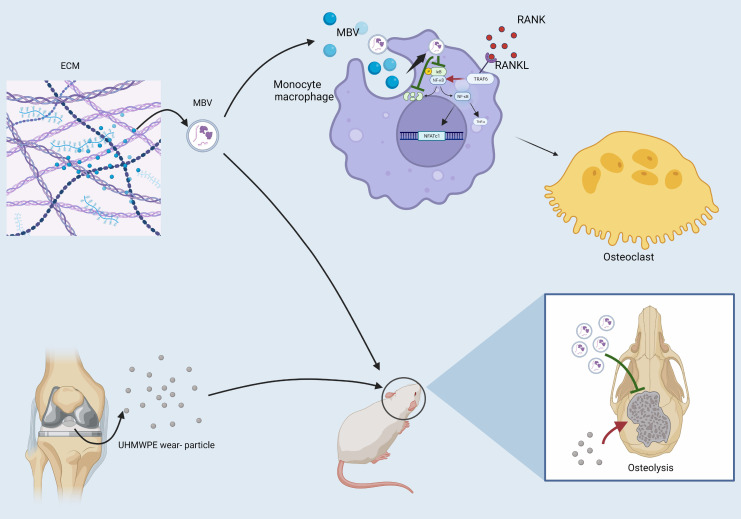
Schematic illustration of the mechanism by which MBVs reduce particulate-induced osteolysis. MBVs were derived from a porcine urinary bladder extracellular matrix (UBM) bioscaffold. In vitro, MBVs reduce monocyte/macrophage differentiation to osteoclast by suppressing the NF-κB pathway and its downstream NFATc1, DC-STAMP, c-Src, and cathepsin K expression. In vivo, MBVs alleviate UHMWPE particle-induced osteolysis in a murine calvarial osteolysis model.

The results of the present study demonstrate that MBV treatment can attenuate RANKL-induced osteoclast formation in vitro, as indicated by reduced TRAP-positive cells and osteoclast activity (Fig. 7). In PPOL, particulate debris released from the prosthesis is phagocytosed by macrophages, resulting in RANKL production, NF-κB pathway activation, and osteoclastogenic gene expression (*5*, *44*, *45*). These monocyte/macrophages differentiate to osteoclast precursor cells, then fuse to form large multinucleated osteoclasts that secrete MMPs and cathepsin K to degrade bone matrix (*33*). Results of the present study indicate that MBV can inhibit the degradation of IκBα to suppress NF-κB–mediated production of TNF-α and downstream osteoclastogenic transcription factors NFATc1 and DC-STAMP, with concomitant decreases in cathepsin K and c-Src (Fig. 7). While more investigation is required to determine the specific molecular components of MBV that interact with IκBα to inhibit its degradation, the finding that MBV can modulate the NF-κB pathway suggests potential applications in other inflammatory diseases. Moreover, MBVs that inhibit NF-κB pathway might reveal UBM NF-κB pathway inhibition ability source (*24*). The results of the present study and other studies delineating the immunomodulatory effects of MBV treatment in vitro and in in vivo models of RA and virus-induced cytokine storm support such a view (*30*, *46*). It has been shown that MBV-treated macrophages up-regulate arginase expression, which is known to disrupt the intracellular TCA cycle and inhibit differentiation to osteoclasts, suggesting a multimodal function in inhibiting osteoclastogenesis (*47*).

The debris released from the prosthesis during PPOL is confined to the joint cavity, causing local inflammation and osteolysis (*48*). Because of the anatomical structure of the joint cavity, systemically administered drugs have limited access to the joint cavity; therefore intra-articular injection is typically required (*49*). Previous studies have shown that MBV can be maintained locally for 7 days following intramuscular and subcutaneous injections in nude mice under physiological conditions without toxicity (*50*). The results of the present study show that following local injection, MBVs persist in the cavity formed between the cranial bone and the overlying skin-muscle layer and maintain a local concentration approximately 20-fold higher than the liver and kidneys. In clinical scenarios of PPOL, the feasibility of knee joint cavity injection makes local delivery a practical option. However, hip joint injection is less straightforward and may require ultrasound guidance, increasing the difficulty of the local administration. Further investigation of routes of administration and dose/dose regimen of MBV is warranted to expand clinical applications (*49*). Another potential strategy is to engineer or modify MBV to target specific cell types. For instance, encapsulation of human urine–derived stem cell (HUSCs) EV with macrophage cell membrane fragments was reported to increase EV uptake by BMSCs, suggesting that similar methods could be applied to MBV (*42*).

Results of the present show that MBV can modulate inflammation to attenuate bone remodeling caused by particle-induced osteolysis while preserving bone mass (Fig. 7). Several studies have outlined a role for other EV types in bone formation and resorption. Matrix vesicles in the growth plate cartilage and bone were the first EV species shown to mediate bone mineralization directly (*51*). Further study showed that bone EV from young mice could induce osteogenesis, while those from old mice instead induced vascular calcification (*52*). Other studies found that tumor-derived exosomes can contribute to dysregulation of bone homeostasis. Specifically, exosomes from metastatic prostate tumors induced osteoclastogenesis and inhibited osteogenesis, whereas HUSCs EV had the opposite effects (*40*, *53*, *54*). Porcine bladder ECM-derived MBV attenuated bone destruction in RA, and it was speculated that the immunomodulatory function of MBV played a major role in these effects (*29*). The current study expands on the regulatory function of MBV and underlines their ability to modulate both inflammation and osteoclast formation. Future studies to compare the composition and cargo of other EV subtypes that have beneficial effects in bone pathologies could pave the way for new therapeutic approaches (*26*). A single dose and route of administration was tested in the present work. Further exploration of the optimal tissue source, route of administration, and dose of MBV is needed. The results of the present study clearly show that MBVs have therapeutic potential for NF-κB pathway–driven inflammatory diseases such as PPOL and that further studies are warranted.

## MATERIALS AND METHODS

### Preparation of acellular urinary bladder ECM

Acellular porcine urinary bladder ECM (UB-ECM) was prepared as previous described (*25*). Commercial porcine urinary bladder (Animal Biotech Industries, Doylestown, PA, USA) was obtained from market-weight animals (approximately 110 kg). The bladder was mechanically processed to remove tunica serosa, tunica muscularis externa, most of the tunica submucosa, and tunica muscularis mucosa, then soaked in 4% ethanol and 0.1% peracetic acid (Enviroguard MP2, Rochester Midland, USA), and put on a shaker for 2 hours at 300 rpm at room temperature. The tissue was extensively rinsed with PBS (pH 7.4) and deionized water to remove luminal urothelial cells leaving the basement membrane intact. The basement membrane was lyophilized as UB-ECM scaffold and milled into powder by a Wiley mill (Thomas Scientific, Swedesboro, NJ, USA) with a #40 mesh screen.

### Isolation and identification of MBV

MBVs were isolated from ECM bioscaffold as performed in prior studies (*25*, *26*, *50*, *55*). UB-ECM powder was digested in a liberase (10 μg/ml; 05401046001, Roche, Germany) with 50 mM tris, 200 mM NaCl, and 5 mM CaCl_2_ on a tube revolver/rotator at 40 rpm for 16 hours. UB-ECM digestion was centrifuged from 500 to 2500*g* at 4°C for 20 min, then 10,000*g* at 4°C for 3 × 30 min. The clarified supernatant was filtered with 0.22-μm filter, then centrifuged (Optima L-90K Ultracentrifuge, Beckman Coulter, Brea, CA, USA) at 100,000*g* for 70 min. The supernatant was discarded and the pellet was resuspended with 1× PBS (pH 7.4). The morphology of MBV was determined by TEM. Particle concentration and distribution were determined using NTA.

### Mouse monocytes/macrophages culture

Mouse monocytes/macrophage cell line RAW 264.7 was obtained from the American Type Culture Collection (ATCC) and cultured following the ATCC guidelines in DMEM/F12 (11320033, Gibco, USA) with 10% fetal bovine serum (S11150, Bio-Techne Sales Corp, USA), and 1% penicillin-streptomycin (SV30010, Hyclon, USA) at 37°C in 5% CO_2_.

### Live/dead staining

The cell cytotoxicity assay was determined by a live-dead staining kit (R37601, Invitrogen, USA) and obtained images by microscope (Zeiss Axio Observer Z1, Germany). RAW 264.7 cells (1 × 10^4^ cells/well) were cultured in a 96-well plate (12556008, Thermo Fisher Scientific, USA) with PBS, 5% DMSO, and serial dilution of MBV ranging from 1.25 × 10^9^ to 1 × 10^10^ particles/ml, MBV versus cells ratio from 1.25 × to 1 × 10^6^ particles/ml per cell for 24 hours. The live-dead cells were counted by CellProfiler (BROAD Institute).

### CCK-8 assay

The cell proliferation assay was determined by cell counting kit 8 (CCK-8; K1018, APExBIO, USA). RAW 264.7 cells (1 × 10^4^ cells/well) were cultured in a 96-well plate (12556008, Thermo Fisher Scientific, USA) with serial dilution of MBV ranging from 1.25 × 10^9^ to 1 × 10^10^ particles/ml, MBV versus cells ratio from 1.25 × 10^5^ to 1 × 10^6^ particles/ml per cell for 24 hours. At 24 hours, all culture wells were added to 10-μl CCK-8 assay in 90-μl culture media incubated at 37°C for 2 hours and determined the absorbance at 450 nm.

### MBV uptake assay

The MBV uptake assay was determined by labeled CFSE (C1157, Thermo Fisher Scientific, USA) MBV and 4′,6-diamidino-2-phenylindole (DAPI)–labeled (0100-20, SouthernBiotech, USA) nuclei under a fluorescence microscope. MBVs (approximately 1 × 10^12^ particles/ml) were cultured with CFSE as 299:1 dilution for 2 hours at 37°C. After incubation, the MBV CFSE mix was diluted by 1× PBS as 1:7 and added to a size exclusion chromatography column to remove residue CFSE. CFSE-labeled MBVs were collected and diluted to 1 × 10^11^ particles/ml by 1× particle-free PBS. RAW 264.7 cells were treated with CFSE-labeled MBV in a μ-slide eight-well ibiTreat plate (80826, Ibidi, Germany) for 1 hour. Then, the cells were fixed by a 4% paraformaldehyde (PFA) and the cells’ nuclei were labeled by DAPI (OB010020, Thermo Fisher Scientific, USA) and F-actin was labeled by Alexa Fluor 594 phalloidin (A12381, Thermo Fisher Scientific, USA), then visualized under a Zeiss AxioObserver Z1 microscope.

### In vitro osteoclast induction of RAW 264.7 monocytes/macrophages

Recombinant RANKL (390-TN, R&D System, USA) was used from 30 to 50 ng/ml for 0 min to 5 days to induce RAW 264.7 cells differentiate into osteoclasts. For osteoclast formation and supernatant TNF-α enzyme-linked immunosorbent assay (ELISA), RAW 264.7 cells were seeded in 48-well plates at a 6.25 × 10^3^cells/cm^2^ density and stimulated with RANKL (30 ng/ml) for 5 days (*56*). For osteoclast-related gene expression determination, RAW 264.7 cells were seeded in six-well plates at a density of 5× 10^5^ cells per well and stimulated with RANKL (50 ng/ml) for 1 day. For osteoclast-related protein expression determination, RAW 264.7 cells were seeded in six-well plates at a density of 1.5 × 10^5^ cells with RANKL (50 ng/ml) stimulation for 3 days. For NF-κB pathway–related Western blot, RAW 264.7 cells were seeded in six-well plates at a density of 1× 10^6^ cells per well with RANKL (50 ng/ml) stimulation from 0 to 60 min.

### TRAP staining

The osteoclasts induced by RANKL from RAW 264.7 cells were determined by tartrate-resistant acid phosphorus staining kit (387A, Sigma-Aldrich, USA) following manufacturer’s instruction. PFA (4%; pH 7.0) was used to fix RANKL-induced RAW 264.7 cells. Fixed cells were washed with 1× PBS and treated with TRAP staining solution for 1 hour at 37°C. Stained cells were washed with 1× PBS twice and stained with hematoxylin then washed under tap water. The TRAP^+^ cells were visualized under a Zeiss AxioObserver Z1 microscope.

### Acting ring staining

RANKL-induced RAW 264.7 cells were fixed by 4% PFA (pH 7.0) and washed with 1× PBS. The osteoclasts actin rings were determined by Alexa Fluor 488 phalloidin (A12379, Thermo Fisher Scientific, USA) following manufacturer’s guideline and DAPI-labeled (0100-20, SouthernBiotech, USA) nuclei. The actin rings and nuclei were visualized under a Zeiss AxioObserver Z1 microscope.

### Bone resorption assay

Following the manufacturer’s instructions, RAW 264.7 cells were seeded in a 48-well fluoresceinamine-labeled chondroitin sulfate calcium phosphate–coated plate (Cosmo Bio, CSR-BRA-48KIT, USA) at a 6.25 × 10^3^cells/cm^2^ density and stimulated with 1 ml of RANKL (30 ng/ml) phenol red-free macrophage/monocyte media for 7 days. At day 6, 100-μl media was moved to a 96-well plate mix with 50 μl of bone resorption buffer (Cosmo Bio, CSR-BRA-48KIT, USA) to assess fluorescence intensity with a plate reader at 485/535 nm. At day 7, the cells were removed with 5% sodium hypochlorite. Resorption pit area was visualized under a Zeiss AxioObserver Z1 microscope.

### Quantitative RT-qPCR

RANKL-induced RAW 264.7 cells were treated with MBV (1× 10^10^ or 2 × 10^10^ particles/ml) at the same time (MBV versus cells ratio 1× 10^4^ to 2 × 10^4^ particles/ml per cell) and generated RNA by a commercial RNA extraction kit (74536, Qiagen, USA). The concentration and purity of total RNA were determined with nanodrop spectrophotometers (NanoDrop 2000, Thermo Fisher Scientific, USA). A reverse transcription kit (18080051, Thermo Fisher Scientific, USA) was used to synthesize cDNA. The gene levels of NFATc1, DC-STAMP, cathepsin K, c-Src, β3-integrin, and MMP-9 were measured by Quant Studio 6 (Thermo Fisher Scientific, USA) using a commercial SYBR mix (A25778, Applied Biosystem, USA) as well as the forward and reverse primers listed in table S1. The GADPH gene was used as the housekeeping gene. The RT-qPCR results were analyzed using delta-delta CT method.

### Western blot

For osteoclast protein determination, RANKL-induced RAW 264.7 cells were treated with MBV (1× 10^10^ or 2 × 10^10^ particles/ml) at the same time. For MBV function on RANKL-activated NF-κB pathway investigation, RAW 264.7 cells were pretreated with MBV (2 × 10^10^ particles/ml) for 2 hours then treated with RANKL from 0 to 60 min. To collect total protein, the plates were placed on ice, washed with cold PBS twice, and lysed with radioimmunoprecipitation buffer (PI89900, Thermo Fisher Scientific, USA) with added protease/phosphatase inhibitor (A32963, Thermo Fisher Scientific, USA). The protein concentration of lysis buffer was quantified by a commercial BCA kit (PI23224, Thermo Fisher Scientific, USA). After determining the total protein concentration, the protein buffer was diluted by a containing 10% β-mercaptoethanol (M3148-100ML, Sigma-Aldrich, USA) 4× loading buffer (1610747, Bio-rad, USA) at a volume ratio of 1:3 then heated to 95°C for 5 min to denature protein. The protein was separated by using 4 to 20% sulfate–polyacrylamide gel electrophoresis (456-1093, Bio-rad, USA) and transferred to polyvinylidene difluoride membranes (1620177, Bio-rad, USA). Then, membranes were blocked with 5% nonfat milk in tris-buffered saline containing 0.1% Tween-20 (TBST) for 1 hour at room temperature, and subsequently probed with 1:1000 dilution primary antibodies NFATc1 (8032S,Cell Signaling Technology, USA), cathepsin K (57056S,Cell Signaling Technology, USA), c-Src (25978-1-AP, Proteintech, USA), phospho-p65 (3033S, Cell Signaling Technology, USA), p65 (8242S, Cell Signaling Technology, USA), IκBα (4812S, Cell Signaling Technology, USA), and β-actin (4970T, Cell Signaling Technology, USA) in TBST and 1% bovine serum albumin (BSA) overnight at 4°C. The probes primary antibody membrane was washed with TBST then incubated with 1:5000 secondary antibody (Sigma-Aldrich, A0545, USA) at room temperature for 60 min, washed with TBST, and then exposed to a chemiluminescent substrate detection reagent (PI34095, Thermo Fisher Scientific, USA) for visualization.

### Enzyme-linked immunosorbent assay

Commercial mouse TNF-α ELISA (MTA00B, R&D Systems, USA) kit was used to determine TNF-α concentration within RANKL-induced osteoclast culture media at days 5 following manufacturer’s instruction.

### UHMWPE particulate-induced mice calvarial osteolysis

The objective of the present study was to investigate the role of local MBV administration in treating mouse pericalvarial particulate-induced osteolysis and periosteal inflammation. A mouse pericalvarial UHMWPE implantation model was used to simulate in vivo prosthetic wear debris–induced osteolysis. BALB/c mice were randomly divided into a SHAM surgery group, UHMWPE + PBS (vehicle) group, and UHMWPE + MBV (treatment) group; each group included five mice. Each mouse was placed in the prone position under anesthesia, hair was removed from the parietal region, the parietal area was disinfected with complex iodine and 70% alcohol, and an incision was made along the median sagittal line of the skull from the midpoint of the eyes to the midpoint of the ears with a scalpel; the galea aponeurotica was incised by extending the incision, the periosteum attached to the bony surface of the skull was removed, and 30 mg of endotoxin-free UHMWPE powder (φ = 5 μm,ceridust 3610, Clariant, Gersthofen, Germany) was uniformly applied to the bony surface of the skull. The muscles and the skin were closed with 4-0 sutures (*57*). Postsurgery, 100 μl (PBS diluted 2 × 10^11^ particle (20 μg) per mouse MBV administrated of MBV (2 × 10^11^ particles in PBS) was administrated to the pericalvarial area within the capacity between the skin-muscle layer and skull of each mouse at days 0, 7, 14, and 21 by local injection. The vehicle group was administrated the same volume PBS. On day 28, mice were euthanized and calvarial caps harvested. The calvarial caps were fixed by PFA (pH 7.4) for 24 hours and then rinsed with PBS (pH 7.4) for micro-CT scanning. High-resolution skull film micro-CT (Scanco μCT 50, Scanco Medical AG, Bassersdorf, Switzerland) was performed with the voxel resolution at 10 μm. The micro-CT slides were three-dimensionally reconstructed by mimics 17.0 (Materialise, Leuven, Belgium). After reconstruction, the region of interest (400 coronal layers) was selected by image Pro Plus 6.0 (Media Cybernetics, Maryland, USA) along the bone boundary to measure bone area and pores area to calculate Ct.Po and BV/TV. The calvarial caps were decalcified in 10% EDTA for 14 days, followed by paraffin sectioning for histology and immunolabeling staining. Animal studies were conducted with the approval of the University of Pittsburgh Institutional Animal Care and Use Committee (protocol 00021619).

### MBV biodistribution in particulate-induced osteolysis model in vivo

Mice were fed an imaging diet (AIN-76A, Bio-Serv, USA) 2 weeks before biodistribution examination to prevent background fluorescence. The UHMWPE particulate-induced mice calvarial osteolysis model was established as described above. MBVs with PBS suspension and same volume PBS were processed by an EV near-IR label kit (EXOGV900A, System Bioscience, USA) following the manufacturer’s introduction. Two days postsurgery, 100 μl of approximately 100-μg near-IR–labeled MBV and dye-only PBS were administrated pericalvarial separately into the MBV and dye-only control groups. Following injection, animals were imaged at 0 hours, 3 hours, 6 hours, and 1, 2, 3, 4, 5, and 7 days using the IVIS Spectrum (*n* = 5 animals; PerkinElmer, USA). At 7 days, mice were euthanized, and calvarial caps, hearts, lungs, livers, kidneys, and lower limb bones were collected to acquire fluorescence images using IVIS Spectrum. Images were processed and quantified using IVIS software (PerkinElmer, USA).

### Histological and Immunolabeling analyses

Paraffin sections were deparaffinized with progressive xylene, followed by ethanol rinses decreasing from 100 to 70%, then running tap water and deionized water rinses to rehydrate sections. Hydrated sections stained with hematoxylin and eosin (H&E) staining and TRAP staining. For immunolabeling, hydrated sections were processed with proteinase K for 30 min to unmask antigen. Endogenous peroxidase and alkaline phosphatase were blocked with 2.5% horse serum. Then the sections were probed with TNF-α primary antibody (1:200, Abcam, ab1793, USA) overnight at 4°C. Primary antibody probes sections were washed with TBST and incubated with amplifier antibody, polymer reagent, and DAB solution (MP-7602, Vector Laboratories, USA) for color reactions. The images were visualized under a light microscope. Macrophage phenotype was assessed by immunofluorescence staining. The same deparaffinized and rehydrated sections were processed with proteinase K for 30 min to unmask the antigen. Then, the sections were blocked with 4% donkey serum, 2% BSA, and 0.1% Triton X-100 TBST buffer at room temperature for 60 min. The 1% BSA-TBST dilute CD86 (1:100, rabbit–anti-mouse, Bioss, BS-1035R, USA) and CD206 (1:100, goat–anti-mouse, R&D Systems, AF2535, USA) primary antibodies probe sections overnight at 4°C. Primary antibody probes sections were rinsed washed with TBST and incubated with fluorescence conjugated secondary antibody (1:500, 488 donkey anti-rabbit, Invitrogen, R37119, USA; and 1:500, 594 donkey anti-goat, Invitrogen, A11055, USA) avoid from light at room temperature for 60 min. Rinsed and was hed sections were immersed with a DAPI mounting media (Southern Biotech, 0100-20, USA) and covered with a coverslip. The CD86 was assigned a red channel, and the CD206 was assigned a green channel. CD86 was assigned to the red channel and CD206 was assigned green channel. The H&E and TRAP staining sections were visualized by MoticEasyScan (Schertz, TX, USA) digital slide scanner and quantified with Image J FIJI (*58*). The immunolabeling staining sections were visualized under a Zeiss AxioObserver Z1 microscope and quantified with QuPath (*59*). The positive cell QuPath analysis pipeline is set with a workflow: line annotation/pixel width and length setting/rectangle annotation analysis area selection/cell detection (blue nuclei, total cells)/positive cell detection [nuclei blue/cytoplasm color (DAB, red, green)]. Samples were then batch processed to identify these characteristics within all samples in an automated fashion without user input.

### Statistical analysis

All data are presented as means ± SD. Comparisons were performed using *t* test, one-way analysis of variance (ANOVA) with Tukey’s post hoc correction with no adjustments made for multiplicity as appropriate, by using GraphPad Prism version 9.0. *P* < 0.05 was considered statistically significant (*P < 0.05, **P < 0.01, and ***P < 0.001).

## References

[1] M. Sloan, A. Premkumar, N. P. Sheth, Projected volume of primary total joint arthroplasty in the U.S., 2014 to 2030. J. Bone Joint Surg. Am. 100, 1455–1460 (2018). 30180053 10.2106/JBJS.17.01617

[2] G. Labek, M. Thaler, W. Janda, M. Agreiter, B. Stöckl, Revision rates after total joint replacement: Cumulative results from worldwide joint register datasets. J. Bone Joint Surg. 93, 293–297 (2011). 10.1302/0301-620X.93B3.25467 21357948

[3] J. J. Jacobs, N. J. Hallab, R. M. Urban, M. A. Wimmer, Wear particles. J. Bone Joint Surg. Am. 88, 99–102 (2006). 16595453 10.2106/JBJS.F.00102

[4] A. M. Kandahari, X. Yang, K. A. Laroche, A. S. Dighe, D. Pan, Q. Cui, A review of UHMWPE wear-induced osteolysis: The role for early detection of the immune response. Bone Res. 4, 16014 (2016). 27468360 10.1038/boneres.2016.14 PMC4941197

[5] J. H. Park, N. K. Lee, S. Y. Lee, Current understanding of RANK signaling in osteoclast differentiation and maturation. Mol. Cells 40, 706–713 (2017). 29047262 10.14348/molcells.2017.0225 PMC5682248

[6] P. Sadoghi, M. Liebensteiner, M. Agreiter, A. Leithner, N. Bohler, G. Labek, Revision surgery after total joint arthroplasty: A complication-based analysis using worldwide arthroplasty registers. J. Arthroplasty 28, 1329–1332 (2013). 23602418 10.1016/j.arth.2013.01.012

[7] G. S. Hussey, J. L. Dziki, S. F. Badylak, Extracellular matrix-based materials for regenerative medicine. Nat. Rev. Mater. 3, 159–173 (2018).

[8] J. D. Naranjo, L. T. Saldin, E. Sobieski, L. M. Quijano, R. C. Hill, P. G. Chan, C. Torres, J. L. Dziki, M. C. Cramer, Y. C. Lee, R. Das, A. K. Bajwa, R. Nossair, M. Klimak, L. Marchal, S. Patel, S. S. Velankar, K. C. Hansen, K. McGrath, S. F. Badylak, Esophageal extracellular matrix hydrogel mitigates metaplastic change in a dog model of Barrett’s esophagus. Sci. Adv. 6, eaba4526 (2020). 32656339 10.1126/sciadv.aba4526 PMC7329334

[9] L. T. Saldin, M. Klimak, R. C. Hill, M. C. Cramer, L. Huleihel, X. Li, M. Quidgley-Martin, D. Cardenas, T. J. Keane, R. Londono, G. Hussey, L. Kelly, J. E. Kosovec, E. J. Lloyd, A. N. Omstead, L. Zhang, A. Nieponice, B. Jobe, K. Hansen, A. H. Zaidi, S. F. Badylak, The effect of normal, metaplastic, and neoplastic esophageal extracellular matrix upon macrophage activation. J. Immunol. Regen. Med. 13, 100037 (2021). 34027260 10.1016/j.regen.2020.100037 PMC8136242

[10] N. Mehrban, C. Pineda Molina, L. M. Quijano, J. Bowen, S. A. Johnson, J. Bartolacci, J. T. Chang, D. A. Scott, D. N. Woolfson, M. A. Birchall, S. F. Badylak, Host macrophage response to injectable hydrogels derived from ECM and α-helical peptides. Acta Biomater. 111, 141–152 (2020). 32447065 10.1016/j.actbio.2020.05.022

[11] B. M. Sicari, J. P. Rubin, C. L. Dearth, M. T. Wolf, F. Ambrosio, M. Boninger, N. J. Turner, D. J. Weber, T. W. Simpson, A. Wyse, E. H. Brown, J. L. Dziki, L. E. Fisher, S. Brown, S. F. Badylak, An acellular biologic scaffold promotes skeletal muscle formation in mice and humans with volumetric muscle loss. Sci. Transl. Med. 6, 234ra258 (2014). 10.1126/scitranslmed.3008085 PMC5942588 24786326

[12] S. F. Badylak, D. O. Freytes, T. W. Gilbert, Reprint of: Extracellular matrix as a biological scaffold material: Structure and function. Acta Biomater. 23, S17–S26 (2015). 26235342 10.1016/j.actbio.2015.07.016

[13] A. D. Theocharis, S. S. Skandalis, C. Gialeli, N. K. Karamanos, Extracellular matrix structure. Adv. Drug Deliv. Rev. 97, 4–27 (2016). 26562801 10.1016/j.addr.2015.11.001

[14] A. Petrosyan, S. Da Sacco, N. Tripuraneni, U. Kreuser, M. Lavarreda-Pearce, R. Tamburrini, R. E. De Filippo, G. Orlando, P. Cravedi, L. Perin, A step towards clinical application of acellular matrix: A clue from macrophage polarization. Matrix Biol. 57–58, 334–346 (2017). 10.1016/j.matbio.2016.08.009 PMC6717660 27575985

[15] T. J. Keane, J. Dziki, E. Sobieski, A. Smoulder, A. Castleton, N. Turner, L. J. White, S. F. Badylak, Restoring mucosal barrier function and modifying macrophage phenotype with an extracellular matrix hydrogel: Potential therapy for ulcerative colitis. J. Crohns Colitis 11, 360–368 (2017). 27543807 10.1093/ecco-jcc/jjw149

[16] L. Huleihel, J. L. Dziki, J. G. Bartolacci, T. Rausch, M. E. Scarritt, M. C. Cramer, T. Vorobyov, S. T. LoPresti, I. T. Swineheart, L. J. White, B. N. Brown, S. F. Badylak, Macrophage phenotype in response to ECM bioscaffolds. Semin. Immunol. 29, 2–13 (2017). 28736160 10.1016/j.smim.2017.04.004 PMC5612880

[17] B. M. Sicari, J. L. Dziki, B. F. Siu, C. J. Medberry, C. L. Dearth, S. F. Badylak, The promotion of a constructive macrophage phenotype by solubilized extracellular matrix. Biomaterials 35, 8605–8612 (2014). 25043569 10.1016/j.biomaterials.2014.06.060

[18] R. Londono, J. L. Dziki, E. Haljasmaa, N. J. Turner, C. A. Leifer, S. F. Badylak, The effect of cell debris within biologic scaffolds upon the macrophage response. J. Biomed. Mater. Res. A 105, 2109–2118 (2017). 28263432 10.1002/jbm.a.36055

[19] F. W. Meng, P. F. Slivka, C. L. Dearth, S. F. Badylak, Solubilized extracellular matrix from brain and urinary bladder elicits distinct functional and phenotypic responses in macrophages. Biomaterials 46, 131–140 (2015). 25678122 10.1016/j.biomaterials.2014.12.044

[20] D. T. Ploeger, S. M. van Putten, J. A. Koerts, M. J. van Luyn, M. C. Harmsen, Human macrophages primed with angiogenic factors show dynamic plasticity, irrespective of extracellular matrix components. Immunobiology 217, 299–306 (2012). 22093249 10.1016/j.imbio.2011.10.007

[21] R. Londono, S. F. Badylak, Biologic scaffolds for regenerative medicine: Mechanisms of in vivo remodeling. Ann. Biomed. Eng. 43, 577–592 (2015). 25213186 10.1007/s10439-014-1103-8

[22] J. L. Dziki, D. S. Wang, C. Pineda, B. M. Sicari, T. Rausch, S. F. Badylak, Solubilized extracellular matrix bioscaffolds derived from diverse source tissues differentially influence macrophage phenotype. J. Biomed. Mater. Res. A 105, 138–147 (2017). 27601305 10.1002/jbm.a.35894

[23] B. N. Brown, R. Londono, S. Tottey, L. Zhang, K. A. Kukla, M. T. Wolf, K. A. Daly, J. E. Reing, S. F. Badylak, Macrophage phenotype as a predictor of constructive remodeling following the implantation of biologically derived surgical mesh materials. Acta Biomater. 8, 978–987 (2012). 22166681 10.1016/j.actbio.2011.11.031 PMC4325370

[24] H. N. Jacobs, S. Rathod, M. T. Wolf, J. H. Elisseeff, Intra-articular injection of urinary bladder matrix reduces osteoarthritis development. AAPS J. 19, 141–149 (2017). 27778194 10.1208/s12248-016-9999-6 PMC6005649

[25] L. Huleihel, G. S. Hussey, J. D. Naranjo, L. Zhang, J. L. Dziki, N. J. Turner, D. B. Stolz, S. F. Badylak, Matrix-bound nanovesicles within ECM bioscaffolds. Sci. Adv. 2, e1600502 (2016). 27386584 10.1126/sciadv.1600502 PMC4928894

[26] G. S. Hussey, C. P. Molina, M. C. Cramer, Y. Y. Tyurina, V. A. Tyurin, Y. C. Lee, S. O. El-Mossier, M. H. Murdock, P. S. Timashev, V. E. Kagan, Lipidomics and RNA sequencing reveal a novel subpopulation of nanovesicle within extracellular matrix biomaterials. Sci. Adv. 6, eaay4361 (2020). 32219161 10.1126/sciadv.aay4361 PMC7083606

[27] L. Huleihel, J. G. Bartolacci, J. L. Dziki, T. Vorobyov, B. Arnold, M. E. Scarritt, C. Pineda Molina, S. T. LoPresti, B. N. Brown, J. D. Naranjo, S. F. Badylak, Matrix-bound nanovesicles recapitulate extracellular matrix effects on macrophage phenotype. Tissue Eng. Part A 23, 1283–1294 (2017). 28580875 10.1089/ten.tea.2017.0102 PMC5689118

[28] Y. van der Merwe, A. E. Faust, E. T. Sakalli, C. C. Westrick, G. Hussey, K. C. Chan, I. P. Conner, V. L. N. Fu, S. F. Badylak, M. B. Steketee, Matrix-bound nanovesicles prevent ischemia-induced retinal ganglion cell axon degeneration and death and preserve visual function. Sci. Rep. 9, 3482 (2019). 30837658 10.1038/s41598-019-39861-4 PMC6400956

[29] R. J. Crum, K. Hall, C. P. Molina, G. S. Hussey, E. Graham, H. Li, S. F. Badylak, Immunomodulatory matrix-bound nanovesicles mitigate acute and chronic pristane-induced rheumatoid arthritis. NPJ Regen. Med. 7, 13 (2022). 35110573 10.1038/s41536-022-00208-9 PMC8810774

[30] R. J. Crum, B. R. Huckestien, G. Dwyer, L. Mathews, D. G. Nascari, G. S. Hussey, H. R. Turnquist, J. F. Alcorn, S. F. Badylak, Mitigation of influenza-mediated inflammation by immunomodulatory matrix-bound nanovesicles. Sci. Adv. 9, eadf9016 (2023). 37205761 10.1126/sciadv.adf9016 PMC10198633

[31] L. M. Piening, R. A. Wachs, Matrix-bound nanovesicles: What are they and what do they do? Cells Tissues Organs 212, 111–123 (2023). 35168230 10.1159/000522575

[32] M. C. Cramer, W. A. D’Angelo, M. J. Dewey, A. M. Manuel, S. J. Mullett, S. G. Wendell, D. Napierala, P. Jiang, S. F. Badylak, Extracellular vesicles present in bone, blood and extracellular matrix have distinctive characteristics and biologic roles. J. Immunol. Regen. Med. 18, 100066 (2022).

[33] J. Kodama, T. Kaito, Osteoclast multinucleation: Review of current literature. Int. J. Mol. Sci. 21, 5685 (2020). 32784443 10.3390/ijms21165685 PMC7461040

[34] J. H. Kim, N. Kim, Regulation of NFATc1 in osteoclast differentiation. J. Bone Metab. 21, 233–241 (2014). 25489571 10.11005/jbm.2014.21.4.233 PMC4255043

[35] R. Dai, Z. Wu, H. Y. Chu, J. Lu, A. Lyu, J. Liu, G. Zhang, Cathepsin K: The action in and beyond bone. Front. Cell Dev. Biol. 8, 433 (2020). 32582709 10.3389/fcell.2020.00433 PMC7287012

[36] K. P. McHugh, K. Hodivala-Dilke, M. H. Zheng, N. Namba, J. Lam, D. Novack, X. Feng, F. P. Ross, R. O. Hynes, S. L. Teitelbaum, Mice lacking β3 integrins are osteosclerotic because of dysfunctional osteoclasts. J. Clin. Invest. 105, 433–440 (2000). 10683372 10.1172/JCI8905 PMC289172

[37] K. B. S. Paiva, J. M. Granjeiro, Matrix metalloproteinases in bone resorption, remodeling, and repair. Prog. Mol. Biol. Transl. Sci. 148, 203–303 (2017). 28662823 10.1016/bs.pmbts.2017.05.001

[38] S. L. Teitelbaum, The osteoclast and its unique cytoskeleton. Ann. N. Y. Acad. Sci. 1240, 14–17 (2011). 22172034 10.1111/j.1749-6632.2011.06283.x

[39] Z. Sun, J. Zeng, W. Wang, X. Jia, Q. Wu, D. Yu, Y. Mao, Magnoflorine suppresses MAPK and NF-κB signaling to prevent inflammatory osteolysis induced by titanium particles in vivo and osteoclastogenesis via rankl in vitro. Front. Pharmacol. 11, 389 (2020). 32300300 10.3389/fphar.2020.00389 PMC7142243

[40] H. Li, X. L. Fan, Y. N. Wang, W. Lu, H. Wang, R. Liao, M. Zeng, J. X. Yang, Y. Hu, J. Xie, Extracellular vesicles from human urine-derived stem cells ameliorate particulate polyethylene-induced osteolysis. Int. J. Nanomedicine 16, 7479–7494 (2021). 34785895 10.2147/IJN.S325646 PMC8579861

[41] B. Pan, Z. Zhang, X. Wu, G. Xian, X. Hu, M. Gu, L. Zheng, X. Li, L. Long, W. Chen, P. Sheng, Macrophage-derived exosomes modulate wear particle-induced osteolysis via miR-3470b targeting TAB3/NF-κB signaling. Bioact. Mater. 26, 181–193 (2023). 36911207 10.1016/j.bioactmat.2023.02.028 PMC9999169

[42] J. Xie, Y. Hu, H. Li, Y. Wang, X. Fan, W. Lu, R. Liao, H. Wang, Y. Cheng, Y. Yang, J. Wang, S. Liang, T. Ma, W. Su, Targeted therapy for peri-prosthetic osteolysis using macrophage membrane-encapsulated human urine-derived stem cell extracellular vesicles. Acta Biomater. 160, 297–310 (2023). 36773884 10.1016/j.actbio.2023.02.003

[43] H. Spece, R. V. Yarbrough, S. M. Kurtz, In vivo performance of vitamin E stabilized polyethylene implants for total hip arthroplasty: A review. J. Arthroplasty 38, 970–979 (2023). 36481286 10.1016/j.arth.2022.11.010

[44] S. B. Goodman, J. Gallo, Periprosthetic osteolysis: Mechanisms, prevention and treatment. J. Clin. Med. 8, 2091 (2019). 31805704 10.3390/jcm8122091 PMC6947309

[45] G. Luo, F. Li, X. Li, Z. G. Wang, B. Zhang, TNF-α and RANKL promote osteoclastogenesis by upregulating RANK via the NF-κB pathway. Mol. Med. Rep. 17, 6605–6611 (2018). 29512766 10.3892/mmr.2018.8698 PMC5928634

[46] T. Lawrence, The nuclear factor NF-κB pathway in inflammation. Cold Spring Harb. Perspect. Biol. 1, a001651 (2009). 20457564 10.1101/cshperspect.a001651 PMC2882124

[47] J. S. Brunner, L. Vulliard, M. Hofmann, M. Kieler, A. Lercher, A. Vogel, M. Russier, J. B. Brüggenthies, M. Kerndl, V. Saferding, B. Niederreiter, A. Junza, A. Frauenstein, C. Scholtysek, Y. Mikami, K. Klavins, G. Krönke, A. Bergthaler, J. J. O’Shea, T. Weichhart, F. Meissner, J. S. Smolen, P. Cheng, O. Yanes, J. Menche, P. J. Murray, O. Sharif, S. Blüml, G. Schabbauer, Environmental arginine controls multinuclear giant cell metabolism and formation. Nat. Commun. 11, 431 (2020). 31969567 10.1038/s41467-020-14285-1 PMC6976629

[48] N. A. Hodges, E. M. Sussman, J. P. Stegemann, Aseptic and septic prosthetic joint loosening: Impact of biomaterial wear on immune cell function, inflammation, and infection. Biomaterials 278, 121127 (2021). 34564034 10.1016/j.biomaterials.2021.121127

[49] A. K. Rastogi, K. W. Davis, A. Ross, H. G. Rosas, Fundamentals of joint injection. AJR Am. J. Roentgenol. 207, 484–494 (2016). 27276101 10.2214/AJR.16.16243

[50] R. J. Crum, H. Capella-Monsonis, J. Chang, M. J. Dewey, B. D. Kolich, K. T. Hall, S. O. El-Mossier, D. G. Nascari, G. S. Hussey, S. F. Badylak, Biocompatibility and biodistribution of matrix-bound nanovesicles in vitro and in vivo. Acta Biomater. 155, 113–122 (2023). 36423817 10.1016/j.actbio.2022.11.026

[51] T. Iwayama, P. Bhongsatiern, M. Takedachi, S. Murakami, Matrix vesicle-mediated mineralization and potential applications. J. Dent. Res. 101, 1554–1562 (2022). 35722955 10.1177/00220345221103145

[52] Z. X. Wang, Z. W. Luo, F. X. Li, J. Cao, S. S. Rao, Y. W. Liu, Y. Y. Wang, G. Q. Zhu, J. S. Gong, J. T. Zou, Q. Wang, Y. J. Tan, Y. Zhang, Y. Hu, Y. Y. Li, H. Yin, X. K. Wang, Z. H. He, L. Ren, Z. Z. Liu, X. K. Hu, L. Q. Yuan, R. Xu, C. Y. Chen, H. Xie, Aged bone matrix-derived extracellular vesicles as a messenger for calcification paradox. Nat. Commun. 13, 1453 (2022). 35304471 10.1038/s41467-022-29191-x PMC8933454

[53] F. Urabe, N. Kosaka, Y. Yamamoto, K. Ito, K. Otsuka, C. Soekmadji, S. Egawa, T. Kimura, T. Ochiya, Metastatic prostate cancer-derived extracellular vesicles facilitate osteoclastogenesis by transferring the CDCP1 protein. J. Extracell. Vesicles 12, e12312 (2023). 36880252 10.1002/jev2.12312 PMC9989745

[54] L. Yu, B. Sui, W. Fan, L. Lei, L. Zhou, L. Yang, Y. Diao, Y. Zhang, Z. Li, J. Liu, X. Hao, Exosomes derived from osteogenic tumor activate osteoclast differentiation and concurrently inhibit osteogenesis by transferring COL1A1-targeting miRNA-92a-1-5p. J. Extracell. Vesicles 10, e12056 (2021). 33489015 10.1002/jev2.12056 PMC7812369

[55] L. M. Quijano, J. D. Naranjo, S. O. El-Mossier, N. J. Turner, C. Pineda Molina, J. Bartolacci, L. Zhang, L. White, H. Li, S. F. Badylak, Matrix-bound nanovesicles: The effects of isolation method upon yield, purity, and function. Tissue Eng. Part C Methods 26, 528–540 (2020). 33012221 10.1089/ten.tec.2020.0243 PMC7869881

[56] C. Song, X. Yang, Y. Lei, Z. Zhang, W. Smith, J. Yan, L. Kong, Evaluation of efficacy on RANKL induced osteoclast from RAW264.7 cells. J. Cell Physiol. 234, 11969–11975 (2019). 30515780 10.1002/jcp.27852

[57] M. von Knoch, C. Wedemeyer, A. Pingsmann, F. von Knoch, G. Hilken, C. Sprecher, F. Henschke, B. Barden, F. Löer, The decrease of particle-induced osteolysis after a single dose of bisphosphonate. Biomaterials 26, 1803–1808 (2005). 15576154 10.1016/j.biomaterials.2004.06.010

[58] J. Schindelin, I. Arganda-Carreras, E. Frise, V. Kaynig, M. Longair, T. Pietzsch, S. Preibisch, C. Rueden, S. Saalfeld, B. Schmid, J. Y. Tinevez, D. J. White, V. Hartenstein, K. Eliceiri, P. Tomancak, A. Cardona, Fiji: An open-source platform for biological-image analysis. Nat. Methods 9, 676–682 (2012). 22743772 10.1038/nmeth.2019 PMC3855844

[59] P. Bankhead, M. B. Loughrey, J. A. Fernández, Y. Dombrowski, D. G. McArt, P. D. Dunne, S. McQuaid, R. T. Gray, L. J. Murray, H. G. Coleman, J. A. James, M. Salto-Tellez, P. W. Hamilton, QuPath: Open source software for digital pathology image analysis. Sci. Rep. 7, 16878 (2017). 29203879 10.1038/s41598-017-17204-5 PMC5715110

