# Supplementary material for: Matrix-bound nanovesicles alleviate particulate-induced periprosthetic osteolysis
Source: Sci Adv. 2024 Oct 18;10(42):eadn1852. doi: 10.1126/sciadv.adn1852 (PMC11488533; doi:10.1126/sciadv.adn1852)
Supplement: Supplementary file 1 — Figs. S1 and S2 Table S1 [file sciadv.adn1852_sm.pdf]

Science Advances
AAAS

Supplementary Materials for

Matrix-bound nanovesicles alleviate particulate-induced periprosthetic osteolysis

Runzhi Liao *et al.*

Corresponding author: Stephen F. Badylak, badylaks@upmc.edu

Sci. Adv. **10**, eadn1852 (2024)
DOI: 10.1126/sciadv.adn1852

This PDF file includes:

Figs. S1 and S2
Table S1

Fig. S1

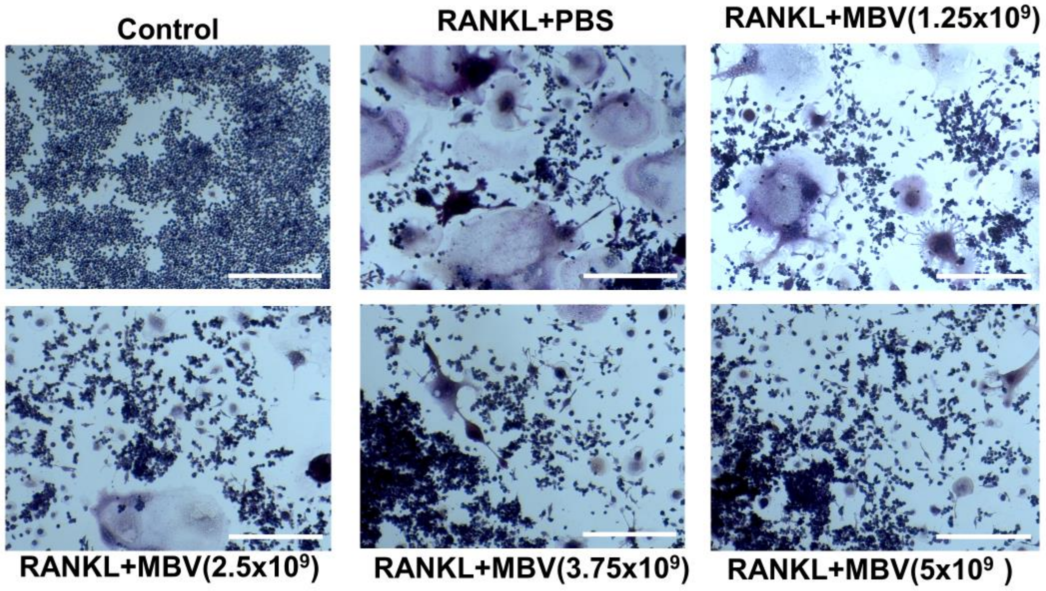

Fig. S1 Osteoclasts tartrate-resistant acid phosphatase (TRAP) staining and Hematoxylin staining. Representative light microscope TRAP staining images (40×). Scale bars 200μm.

Fig. S2

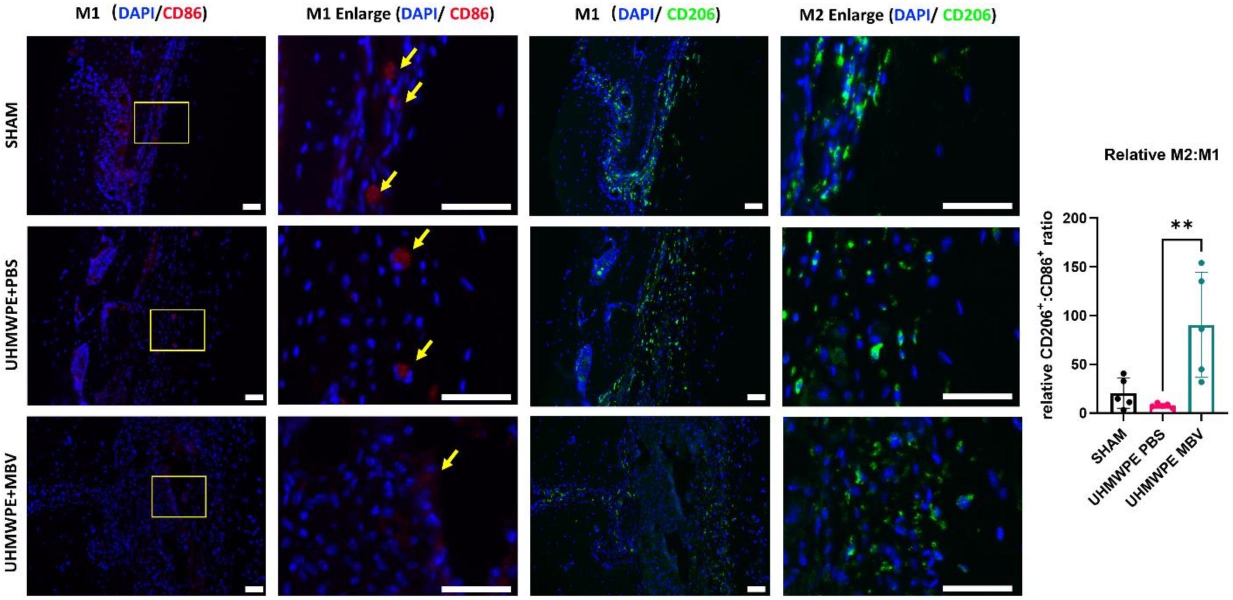

Figure S2. Immunofluorescent staining of the mice calvarial bone coronal paraffin sections for M1-like cells (CD86+) in the left column and M2-like cells (CD206+) in the right column. Scale bar = 50 μm. Values are shown as mean ± SD (n=5); significant differences *P < 0.05.

Table.S1

Oligo name	Oligo sequence (5' to 3')
mouse NFATc1 F	GGGTCAGTGTGACCGAAGAT
mouse NFATc1 R	GGAAGTCAGAAGTGGGTGGA
mouse c-Src F	CCAGGCTGAGGAGTGGTACT
mouse c-Src R	CAGCTTGCGGATCTTGTAGT
mouse β3-Integrin F	TGACATCGAGCAGGTGAAAG
mouse β3-Integrin R	GAGTAGCAAGGCCAATGAGC
mouse Cathepsin K F	GGCCAACTCAAGAAGAAAAC
mouse Cathepsin K R	GTGCTTGCTTCCCTTCTGG
mouse MMP-9 F	AGTTTGGTGTCGCGGAGCAC
mouse MMP-9 R	TACATGAGCGCTTCCGGCAC
mouse GAPDH F	AACTTTGGCATTGTGGAAGG
mouse GAPDH R	ACACATTGGGGGTAGGAACA
mouse DCSTAMP F	TCCTCCATGAACAAACAGTTCCAA
mouse DCSTAMP R	AGACGTGGTTTAGGAATGCAGCTC
